# How Can a High-Performance Screening Strategy Be Determined for Cervical Cancer Prevention? Evidence From a Hierarchical Clustering Analysis of a Multicentric Clinical Study

**DOI:** 10.3389/fonc.2022.816789

**Published:** 2022-01-27

**Authors:** Heling Bao, Xiaosong Zhang, Hui Bi, Yun Zhao, Liwen Fang, Haijun Wang, Linhong Wang

**Affiliations:** ^1^ Department of Maternal and Child Health, Maternal and Child Health Department, School of Public Health, Peking University, Beijing, China; ^2^ National Center for Chronic and Non-communicable Disease Control and Prevention, Chinese Center for Disease Control and Prevention, Beijing, China; ^3^ Department of Obstetrics and Gynecology, Peking University First Hospital, Beijing, China; ^4^ Department of Obstetrics and Gynecology, Peking University People’s Hospital, Beijing, China

**Keywords:** cervical cancer, human papillomavirus, cervical intraepithelial neoplasia, cytology, screening, strategy, cluster analysis, observational study

## Abstract

**Background:**

This study aimed to explore the cluster patterns of cervical cancer screening strategies for detecting high-grade precancerous lesions in terms of benefits, costs, and efficiency.

**Methods:**

A total of 2,065 referral women aged 25–64 years were recruited and underwent human papillomavirus (HPV) testing, liquid-based cytology with manual reading, and cytology with artificial intelligence (AI)-assisted reading. All women were assessed by colposcopy and histological examination. We formed 14 screening strategies based on primary cytology screening, primary HPV screening incorporating HPV-16/18 genotyping triage, cytology triage, or both, and co-testing. The primary outcomes were cervical intraepithelial neoplasia grade 2 or worse (CIN2+) and grade 3 or worse (CIN3+). The hierarchical clustering method was applied to multifaceted indicators, and then, the resulting clusters were described in terms of benefits, costs, efficiency, and their interaction. This study was registered (No. ChiCTR2000034131).

**Results:**

The relative sensitivity of HPV-based strategies compared with cytology alone with the threshold of LSIL+ ranged from 0.68 to 1.19 for CIN2+ detection and from 0.72 to 1.11 for CIN3+ detection, whereas the relative specificity ranged from 0.55 to 1.43 for CIN2+ detection and from 0.51 to 1.51 for CIN3+ detection. Five significant clusters according to the trade-off among benefits, costs, and efficiency were identified. The cluster including four primary HPV screening strategies showed the optimal balance. HPV testing with HPV-16/18 genotyping and AI-based cytology triage presented the optimal trade-off for CIN3+ detection relative to cytology alone in terms of relative sensitivity (1.06), relative specificity (0.72), colposcopies for 1 CIN3+ (3.7 vs. 3.1), a load of follow-up for women with HPV-positive and normal cytology (7.0% vs. 22.3%), and the work of manual cytology reading (35.1% vs. 100%).

**Conclusions:**

Our study provided clinical and methodological evidence on the choice of HPV-based screening strategies. The cluster including primary HPV screening with genotyping and cytology triage showed an optimal balance among benefit, cost, and efficiency.

## Introduction

The World Health Organization (WHO) recommends high-performance cervical cancer screening for women by the age of 35 years old and again at 45 years (1). After that, new cervical cancer screening guidelines issued by the WHO highly recommend human papillomavirus (HPV) testing as primary screening in the general population (2). To date, there has been strong evidence to support the efficacy and effectiveness of primary HPV screening (3, 4). The American Cancer Society (ACS)/American Society for Colposcopy and Cervical Pathology (ASCCP) (5, 6), the American College of Obstetricians and Gynecologists (ACOG) (7), and the US Preventative Services Task Force (USPSTF) (8) also recommend primary HPV screening as the preferred strategy rather than cytology or co-testing. These changes would trigger a switch from conventional screening with cytology or visual inspection with acetic acid (VIA) to primary HPV screening worldwide.

Genital HPV infection is more common (approximately 12%) than the occurrence of cervical intraepithelial neoplasia (CIN) grade 2 or worse (<1%) in the general population (4, 9–12). This means that HPV testing incorporating an appropriate triage is necessary for the high performance of primary HPV screening. Although the “screen, triage, and treat approach” in the WHO guidelines proposes that the benefits and costs of different triage methods are similar (2), there is no universal strategy for all populations, since the performance of HPV-based screening will be affected by many factors, e.g., health resources, HPV prevalence, the feasibility of the method, and quality assurance. Many studies endeavored to reach the trade-off between the benefits and costs of different HPV-based strategies and demonstrated that maximizing sensitivity would inevitably produce relatively poor specificity and require more colposcopies (13–15). However, it remains a methodological question how to determine a high-performance HPV-based strategy for cervical cancer prevention in the locality, especially for resource-limited areas that do not yet adopt an HPV-based strategy.

The study aims to apply clustering techniques to explore cluster patterns of common screening strategies for the detection of high-grade precancerous lesions. It also aims to test the usefulness of unsupervised clustering methods in understanding the integrated performance of different screening strategies and to determine the appropriate cluster to meet different demands for cervical cancer prevention.

## Materials and Methods

### Study Participants and Design

The study enrolled women aged 25–64 years between March 13, 2017 and October 20, 2018. The details of the study design were described previously (16). Briefly, to confirm the diagnosis for each woman and estimate the sensitivity accurately, we selected referral women who had an intact cervix and had no history of CIN and had self-reported genital symptoms in the cervix, suspected cervical invasive cancer on pelvic examination, or any abnormality in organized or opportunistic screening. All eligible participants underwent high-risk HPV testing (Cobas 4800, Roche, USA) and liquid-based cytology (ThinPrep, Bedford, USA) with manual reading. According to our previous work in 2020 (16), artificial intelligence (AI)-assisted cytology (Landing, Wuhan, China) for the detection of cervical intraepithelial neoplasia or invasive cancer was also introduced in this study. Liquid-based slides were automatedly classified as negative and positive results. A panel of cytotechnicians further classified the positive slides according to the Bethesda System (TBS). Manual reading and AI-assisted cytology were conducted in two independent laboratories, and the HPV infection status was masked to both. Each woman was scheduled for immediate colposcopy, and then colposcopy-directed biopsy was performed. Four-quadrant biopsy at the squamocolumnar junction and endocervical curettage were required for all women. Two pathologists from local hospitals performed the histopathological examination, and a pathologist reviewed the results.

The protocol was approved by the ethical committee of the National Center for Chronic and Non-communicable Disease Control and Prevention. All women provided written informed consent before undergoing the study procedures. This observational study was registered (No. ChiCTR2000034131).

### Screening Strategies

**Figure 1:**
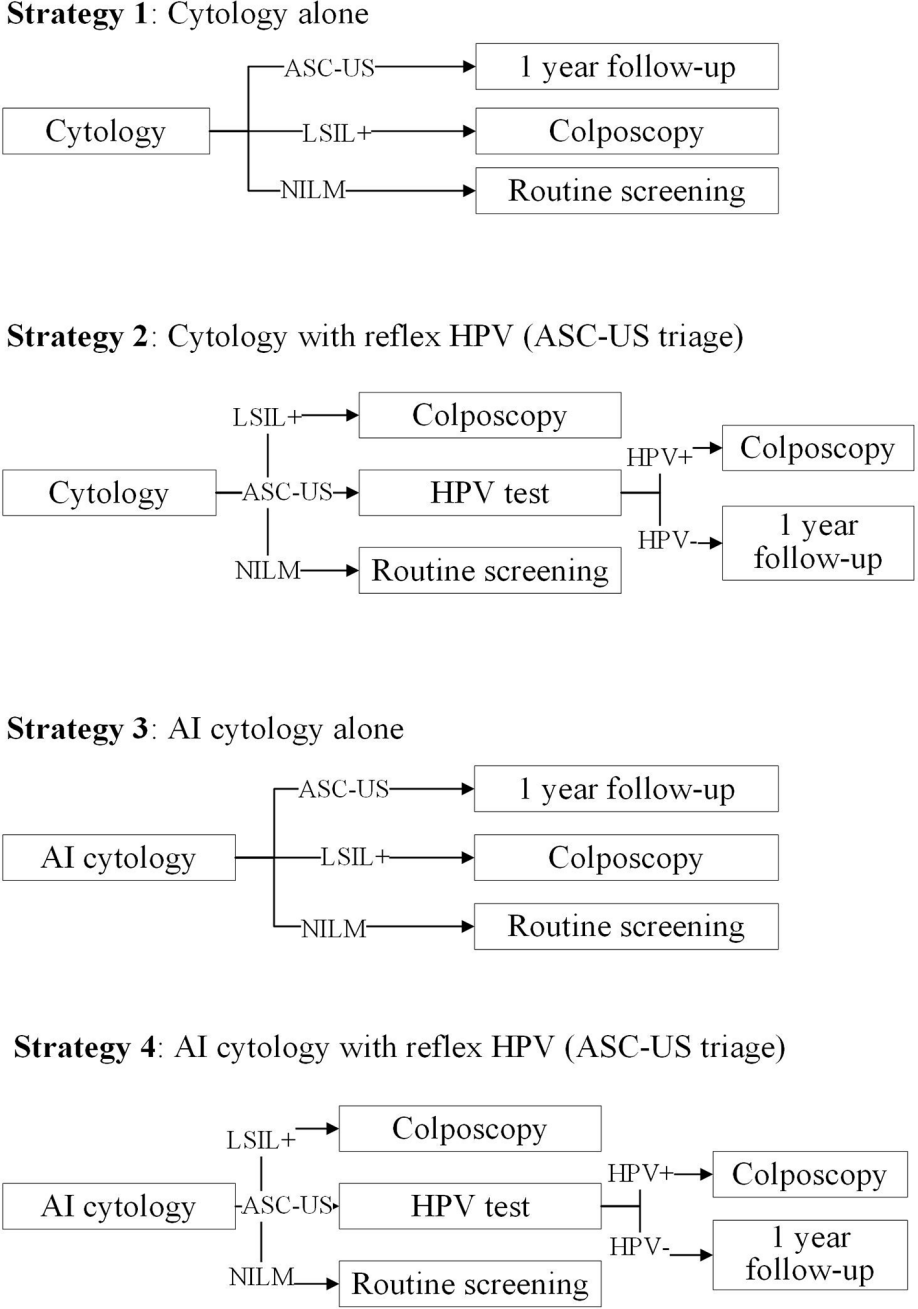
Primary cytology screening strategies in the study. ASC-US, atypical squamous cells of undetermined significance; LSIL, low-grade squamous intraepithelial lesion; HPV, human papillomavirus; AI, artificial intelligence.

**Figure 2:**
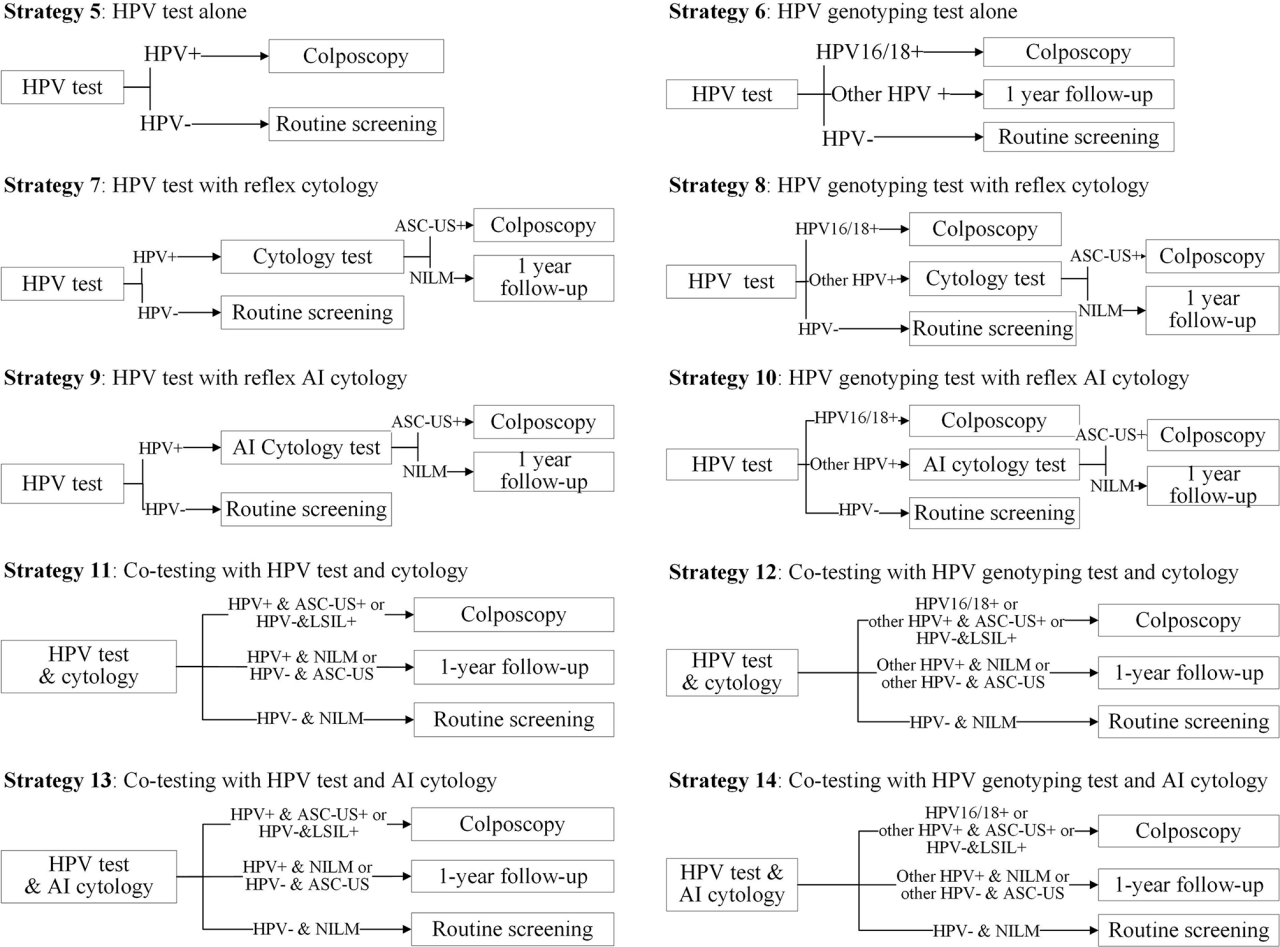
Primary HPV screening strategies and co-testing strategies in the study. HPV, human papillomavirus; ASC-US, atypical squamous cells of undetermined significance; LSIL, low-grade squamous intraepithelial lesion; AI, artificial intelligence.

We formed 14 common screening strategies based on the published literature on cervical cancer screening and those preferred to be chosen by clinicians or policymakers. Strategies 1–4 were cytology-based screening strategies (**Figure 1**). Strategy 1 was cytology alone, referring all women with a low-grade squamous intraepithelial lesion or worse (LSIL+) to immediate colposcopy. Strategy 2 was primary cytology screening with reflex HPV testing, in which women with ASC-US and HPV positive or LSIL+ were referred for immediate colposcopy. Strategies 3 and 4 replaced cytology screening with a manual reading by AI-assisted cytology. Strategies 5–14 were HPV-based screening strategies (**Figure 2**). Strategy 5 was HPV testing alone without triage, and strategies 6–8 were primary HPV screening incorporating HPV16/18 genotyping, reflex cytology, or both. In these strategies, women who did not need immediate colposcopy required repeat testing after 12 months. Strategies 11 and 12 were co-testing with both cytology and HPV testing, incorporating HPV16/18 genotyping or not. Additionally, strategies 9, 10, 13, and 14 used AI-assisted cytology for the triage of HPV-positive women or co-testing. We used cytology alone with the threshold of LSIL+ as a reference for the other 13 strategies.

### Outcomes and Measures

The main outcomes were cervical intraepithelial neoplasia grade 2 or worse (CIN2+) and grade 3 or worse (CIN3+). The study evaluated the screening strategies using three categories of effect indicators. In the category of accuracy, we estimated crude sensitivity and specificity for the detection of CIN2+ and CIN3+, and the relative sensitivity and specificity compared with strategy 1. Given that the high prevalence of outcomes would affect the estimation of sensitivity and specificity, we used the positive likelihood ratio (PLR) and negative likelihood ratio (NLR) as surrogates of accuracy. These indicators represented the ability of testing and would not be affected by the prevalence (17). A higher PLR (more than 1) and lower NLR (close to 0) showed a high performance in distinguishing true positivity and true negativity, respectively. In terms of cost, we calculated the number of tests performed in the screening (including primary testing, reflex testing, and deferred testing), the proportion of manual cytology reading work in the primary and triage tests, and the number of immediate colposcopies. In terms of efficiency, we calculated the number of colposcopies required to detect 1 case.

### Statistical Analysis

In the calculation of crude sensitivity and specificity, Clopper was used to estimate the 95% confidential interval (CI). Relative sensitivity and specificity were estimated with methods described by Kitchener et al. (18). To evaluate the integrated performance of 14 strategies in terms of benefit, cost, and efficiency, we used hierarchical clustering with Ward’s minimum variance method on these indicators. All indicators adopted in the model were centered, and Euclidean distance was used for clustering. Strategies with similar distances are categorized into the same cluster. The average distance within each cluster for indicators showed a better balance when they were close to zero. After that, we displayed 14 strategies in a two-dimensional space between accuracy and cost or efficiency. In the axis of the accuracy, sensitivity was chosen as the key indicator because it was most likely to be considered potentially attractive by policymakers. In the axis of cost and efficiency, the number of colposcopies, colposcopies for detecting one case, the number of tests performed, and the proportion of intensified screening were chosen as key indicators showing the adaptability for different circumstances.

All statistical tests were two-sided, and a *p-*value <0.05 was considered statistically significant. All analyses were conducted with SAS version 9.4 or R package 3.5.1.

## Results

A total of 2,065 women aged 25–64 years were included, and the characteristics of the women are shown in **Table 1**. The mean age was 38.5 years, with most participants at a younger age. Most women were non-smokers and did not have a family history of cancer. Among them, 1,653 (78%) had high-risk HPV infections, and 730 (35.4%) were HPV-16/18 positive. Among 1,660 women with abnormal cytology, there were 460 ASC-US, 517 LSIL, 205 ASC-H, and 478 HSIL or worse. By comparison, AI-assisted cytology automatedly classified 1,588 women as the abnormal group. Finally, there were 390 CIN2, 361 CIN3, and 55 invasive cancers.

**Table 1:** Demographic and clinical characteristics of participants in the study.

	Eligible subjects (n = 2,065)
Age	
Mean (SD)	38.5 (6.5)
25–44, n (%)	1,656 (80.2)
45–64, n (%)	409 (19.8)
Smoking, n (%)	
Current smoker	89 (4.3)
Former smoker	47 (2.3)
Family history of cancer, n (%)	338 (16.4)
HPV infection, n (%)	
HPV positive	1,610 (78.0)
HPV-16/18 positive	730 (35.4)
Non-HPV-16/18 positive	880 (42.6)
Cytology results, n (%)	
ASC-US+	1,660 (80.4)
ASC-US	460 (22.3)
LSIL	517 (25.0)
ASC-H	205 (9.9)
HSIL	437 (21.2)
SCC	33 (1.6)
AGC	8 (0.4)
AI-assisted cytology results, n (%)	
ASC-US+	1,588 (76.9)
ASC-US	565 (27.4)
LSIL	520 (25.2)
ASC-H	239 (11.6)
HSIL	259 (12.5)
AGC	5 (0.2)
Histological results, n (%)	
Negative	535 (27.5)
CIN1	724 (35.1)
CIN2	390 (18.9)
CIN3	361 (17.5)
Cancer	55 (2.7)

HPV, human papillomavirus; ASC-US, atypical squamous cells of undetermined significance; LSIL, low-grade squamous intraepithelial lesion; ASC-H, atypical squamous cells - cannot exclude high-grade squamous intraepithelial lesion; HSIL, high-grade squamous intraepithelial lesion; SCC, squamous cell carcinoma; AGC, atypical glandular cells; CIN, cervical intraepithelial neoplasia; AI, artificial intelligence.

**Tables 2**, **3** show the performance of different screening strategies for the detection of CIN2+ and CIN3+ in the referral population. Strategies 1, 3, and 5 used the positive result of initial testing to determine immediate colposcopy referral. HPV testing alone that referred all HPV-positive women to colposcopy (strategy 5) was more sensitive than cytology alone (strategy 1), detecting 92.4% of CIN2+ cases and 94.0% of CIN3+ cases. However, it had the lowest relative specificity for CIN2+ and CIN3+. Furthermore, this strategy required both the greatest number of colposcopies and the number of colposcopies to detect 1 CIN2+ or CIN3+. HPV testing with 16/18 genotyping triage (strategy 6) substantially decreased the sensitivity, since approximately 37% of CIN3+ cases were associated with non-HPV-16/18 genotypes. It also required the most follow-up work (42.6%) for women who were positive for non-HPV-16/18 types after 12 months.

**Table 2:** Clinical performance of different screening strategies for the detection of CIN2+.

No.	Strategy	Number of tests	Number of colposcopies	Intensified screening,%	Manual reading,%	Colposcopies to detect 1 CIN2+	Sensitivity,%	Relative Sensitivity, 95%CI	Relative specificity, 95%CI	PLR,95%CI	NLR,95%CI
1	Cytology alone^*^	2,065	1,200	22.3	100	1.6	80.8	Reference	Reference	1.75	0.36
2	Cytology with reflex HPV	2,525	1,521	6.7	100	2.1	91.5	1.13 (1.10–1.16)	0.64 (0.60–0.68)	1.39	0.25
3	AI alone^*^	2,065	1,023	27.4	78.5	1.3	79.8	0.99 (0.96–1.04)	1.26 (1.20–1.32)	2.52	0.30
4	AI with reflex HPV	2,630	1,416	8.3	78.5	1.9	93.2	1.16 (1.12–1.20)	0.82 (0.78–0.88)	1.69	0.15
5	HPV alone	2,065	1,610	0	0	2.2	92.4	1.15 (1.11–1.19)	0.57 (0.52–0.62)	1.35	0.24
6	HPV-16/18 alone	2,945	730	42.6	0	1.6	54.8	0.68 (0.64–0.73)	1.43 (1.35–1.52)	2.40	0.59
7	HPV with reflex cytology^¶^	3,790	1,396	8.8	78.0	2.0	87.7	1.09 (1.06–1.12)	0.79 (0.74–0.84)	1.56	0.28
8	HPV with 16/18 genotyping and cytology^¶^	3,025	1,492	4.8	42.6	2.0	90.8	1.13 (1.09–1.17)	0.70 (0.65–0.75)	1.48	0.24
9	HPV with reflex AI cytology	3,910	1,319	12.7	64.8	1.9	88.1	1.09 (1.06–1.14)	0.92 (0.87–0.98)	1.77	0.24
10	HPV with 16/18 genotyping and AI cytology^¶^	3,074	1,449	7.0	35.1	2.0	91.2	1.13 (1.09–1.17)	0.78 (0.72–0.83)	1.59	0.21
11	Co-testing with cytology^¶^	4,450	1,521	15.5	100	2.1	91.5	1.13 (1.10–1.16)	0.64 (0.60–0.68)	1.39	0.25
12	Co-testing with 16/18 genotyping and cytology^¶^	4,368	1,617	11.5	100	2.1	94.5	1.17 (1.14–1.20)	0.55 (0.51–0.59)	1.34	0.19
13	Co-testing with AI cytology^¶^	4,565	1,416	21.1	78.5	1.9	93.2	1.16 (1.12–1.20)	0.82 (0.78–0.88)	1.69	0.15
14	Co-testing with 16/18 genotyping and AI cytology^¶^	4,447	1,546	15.4	78.5	2.0	96.3	1.19 (1.15–1.24)	0.68 (0.63–0.73)	1.53	0.10

^*^Threshold of LSIL.

^¶^Threshold of ASC-US.

CIN, cervical intraepithelial neoplasia; HPV, human papillomavirus; AI, artificial intelligence; PLR, positive likelihood ratio; NLR, negative likelihood ratio; ASC-US, atypical squamous cells of undetermined significance; LSIL, low-grade squamous intraepithelial lesion.

**Table 3:** Clinical performance of different screening strategies for the detection of CIN3+.

No.	Strategy	Test performed	Number of colposcopies	Intensified screening,%	Manualreading,%	Colposcopies to detect 1 CIN3+	Sensitivity,%	Relative sensitivity	Relative specificity	PLR, 95%CI	NLR, 95%CI
1	Cytology alone^*^	2,065	1,200	22.3	100	3.1	87.3	Reference	Reference	1.65	0.27
2	Cytology with reflex HPV	2,525	1,521	6.7	100	3.9	93.7	1.07 (1.04–1.10)	0.61 (0.57–0.64)	1.31	0.22
3	AI alone^*^	2,065	1,023	27.4	78.5	2.6	82.6	0.95 (0.91–0.99)	1.20 (1.15–1.26)	1.94	0.30
4	AI with reflex HPV	2,630	1,416	8.3	78.5	3.7	93.6	1.07 (1.03–1.11)	0.75 (0.70–0.80)	1.46	0.18
5	HPV alone	2,065	1,610	0	0	4.1	94.0	1.08 (1.04–1.12)	0.54 (0.49–0.58)	1.27	0.23
6	HPV-16/18 alone	2,945	730	42.6	0	2.8	63.0	0.72 (0.67–0.78)	1.51 (1.43–1.60)	2.22	0.52
7	HPV with reflex cytology^¶^	3,790	1,396	8.8	78.0	3.7	90.5	1.04 (1.01–1.07)	0.76 (0.71–0.81)	1.43	0.26
8	HPV with 16/18 genotyping and cytology^¶^	3,025	1,492	4.8	42.6	3.8	93.2	1.07 (1.03–1.11)	0.66 (0.62–0.71)	1.38	0.21
9	HPV with reflex AI cytology	3,910	1,319	12.7	64.8	3.6	89.5	1.03 (0.98–1.07)	0.86 (0.81–0.91)	1.53	0.25
10	HPV with 16/18 genotyping and AI cytology^¶^	3,074	1,449	7.0	35.1	3.7	92.8	1.06 (1.02–1.11)	0.72 (0.68–0.77)	1.42	0.21
11	Co-testing with cytology^¶^	4,450	1,521	15.5	100	3.9	93.7	1.07 (1.04–1.10)	0.61 (0.57–0.64)	1.31	0.22
12	Co-testing with 16/8 genotyping and cytology^¶^	4,368	1,617	11.5	100	4.0	96.4	1.10 (107–1.14)	0.51 (0.48–0.55)	1.27	0.15
13	Co-testing with AI cytology^¶^	4,565	1,416	21.1	78.5	3.7	93.6	1.07 (1.03–1.11)	0.75 (0.70–0.80)	1.46	0.18
14	Co-testing with 16/18 genotyping and AI cytology^¶^	4,447	1,546	15.4	78.5	3.8	96.8	1.11 (1.07–1.15)	0.61 (0.56–0.65)	1.37	0.11

^*^Threshold of LSIL.

^¶^Threshold of ASC-US.

CIN, cervical intraepithelial neoplasia; HPV, human papillomavirus; AI, artificial intelligence; PLR, positive likelihood ratio; NLR, negative likelihood ratio; ASC-US, atypical squamous cells of undetermined significance; LSIL, low-grade squamous intraepithelial lesion.

Primary HPV screening followed by reflex cytology (strategy 7) increased the sensitivity for CIN3+ by 4% and required 16% more colposcopies than cytology alone, but only approximately 9% of women required follow-up after 12 months. In contrast, incorporating HPV-16/18 genotyping and cytology triage (strategy 8) could increase the sensitivity for CIN3+ by 7% but require 24% more colposcopies compared with strategy 1. Nonetheless, strategy 8 decreased the proportion of women who required follow-up by 78% compared with strategy 1.

Co-testing incorporating HPV-16/18 genotyping (strategy 12) was the most sensitive screening strategy, detecting 94.5% CIN2+ and 96.4% CIN3+. Co-testing without genotyping triage (strategy 11) only determined colposcopy referral using the results of HPV and reflex cytology, and hence, it had identical performance to strategy 2. Given the utilization of medical resources, the co-testing strategies approximately doubled the number of screening tests in the initial round of screening and increased the colposcopies by 30% compared with strategy 1 and, furthermore, deferred 10–15% of HPV-positive women to 12-month follow-up, which was more than that in primary HPV screening strategies.

Compared with cytology with manual reading, introducing AI-assisted cytology in the primary screening or triage provided comparable sensitivity and higher specificity for CIN2+ and CIN3+. Moreover, AI-assisted cytology classification reduced the manual operations by at least 20% regardless of primary cytology screening or reflex cytology. For example, strategies 9 and 10 had similar sensitivity, higher specificity, and less manual work compared with strategies 7 and 8, respectively.

**Figure 3:**
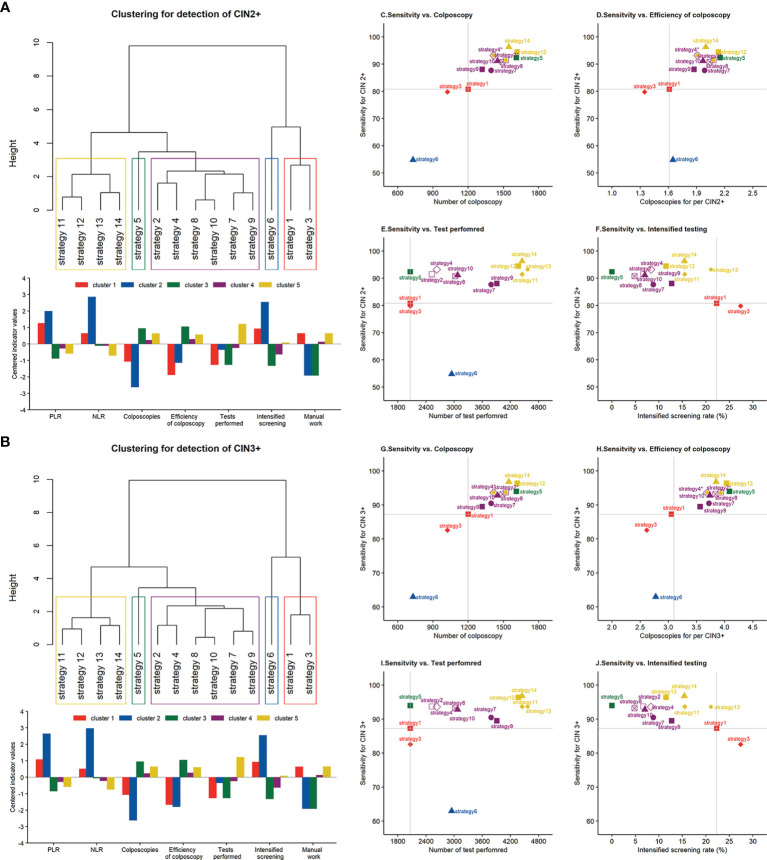
Clustering dendrogram and characterization of different screening strategies for the detection of **(A)** CIN2+ and **(B)** CIN3+. Scatterplot between the sensitivity for CIN2+ and **(C)** the number of colposcopies, **(D)** colposcopies to detect 1 case, **(E)** the number of tests performed, and **(F)** intensified screening rate. Scatterplot between the sensitivity for CIN3+ and **(G)** the number of colposcopies, **(H)** colposcopies to detect 1 case, **(I)** the number of tests performed, and **(J)** intensified screening rate. Note: *strategies 2 and 11, strategies 4 and 13 are at the same coordinates. PLR, positive likelihood ratio; NLR, negative likelihood ratio; CIN, cervical intraepithelial neoplasia.

**Figures 3A, B** show the clustering structure of 14 screening strategies as dendrograms. Five similar clusters were identified when targeting CIN2+ and CIN3+. Clusters 3–5 are nested within a larger cluster that was distinct from clusters 1 and 2, suggesting that there were great variations in terms of accuracy, cost, and efficiency between clusters 1 and 2 and the remaining clusters. Cluster 1, containing the two cytology strategies without any triage, has average colposcopies, relatively higher PLR, and lower NLR compared with other clusters except for cluster 2. Although cluster 2 required the least colposcopies and had the highest PLR, it had the worst NLR compared with other clusters, indicating the lowest probability of true-negative women at the baseline screening. HPV alone fell into an independent cluster (cluster 3), which required the greatest number of colposcopies and colposcopies for detecting 1 case. This suggested that HPV alone had a different pattern of performance in clusters 4 and 5. Cluster 4, containing four primary HPV strategies and two cytology with triage strategies, had average PLR and colposcopies to detect one case and slightly above-average level in terms of NLR and a total number of colposcopies. Cluster 5, containing four co-testing strategies, approximately doubled the number of screening tests compared with the remaining clusters. Because it required 50% more colposcopies than cytology alone, the colposcopies to detect one case were also increased. However, the option had the best NLR, indicating that it would leave out the fewest false-negative women.

**Figures 3C–J** display the trade-off between sensitivity and cost or efficiency indicators for each strategy to understand the balance and utility in routine screening. The analysis displayed the coordinates of each strategy on a diagram where the y-axis always used sensitivity to represent benefit and the x-axis used different indicators to represent costs, such as the number of colposcopies, tests performed, and intensified screening rate. A balanced strategy should provide as high a sensitivity as possible accompanied by moderate consumption. Among these diagrams, strategies 12–14 were the most sensitive and consumed, indicating the inapplicability of co-testing in the general population. By comparison, strategies 8 and 10 displayed similar sensitivity, while their consumption in terms of colposcopy, tests, and follow-up work was moderate.

## Discussion

This study identified five significant clusters from 14 cervical cancer screening strategies in terms of accuracy, cost, and efficiency using hierarchical clustering methods. These clusters are different groupings from the strategy classifications commonly used (13–15). This suggests that hierarchical clustering methods offer an alternative way to synthetically assess screening strategies based on multiple indicator systems. The identification of clusters that share similar patterns of performance may help health decision-makers choose an appropriately high-performance strategy for local cervical cancer prevention and the affordability of health resources. For example, the current screening strategy in a population could be easily transferred to other clusters or another strategy within the same cluster, as these strategies may share a similar benefit–cost ratio and the costs of health resources.

Clusters 3–5 were similar in terms of accuracy, the number of colposcopies, and efficiency. This supports the WHO recommendation that HPV testing should be used as primary screening either with triage or without triage. Nonetheless, cluster 3 (HPV testing alone without triage) had the lowest PLR and efficiency of colposcopy, indicating the necessity of a triage option for HPV-positive women. Furthermore, different triage strategies have divergent patterns of benefit–cost ratios and requirements for local resources, although they were nested in the same cluster. Hence, it is important to consider the requirements and necessary elements underlying the specific strategy, not only the accuracy indicators.

Although previous studies have evaluated the performance of different screening strategies and considered the balance between benefits and harms (13–15), this study focused on the clustering pattern of these strategies based on not only sensitivity and colposcopy but also the efficiency of colposcopy, the load of follow-up, and the work of manual reading. The results presented here may help to explain the pattern of how to direct the choice of screening strategy locally. For example, for some regions that did not establish a management system to follow women with HPV positivity and normal cytology, HPV testing with reflex cytology would not be optimal because loss to follow-up of these women would negate the benefits of primary HPV screening (19). Although co-testing strategies provide the highest sensitivity and a longer interval of repeat screening (20, 21), they require the largest number of initial and intensified tests.

HPV testing alone is convenient and effective (22), but in our opinion, it is necessary to implement a triage method on HPV-positive women to reduce the potential harms (23). Consistent with previous studies (13–15), the combination of 16/18 genotyping and reflex cytology was the optimal strategy for HPV-positive women in our study. It not only achieved similar sensitivity compared with co-testing but also substantially decreased the number of initial and intensified tests and the load of follow-up. Nonetheless, HPV-16/18 genotyping alone (strategy 5) is unadvisable because it would miss approximately 37% of CIN3+ cases. Primary cytology screening with HPV triage (strategies 2 and 4) was nested in the same cluster as primary HPV screening (strategies 7–10), showing a similar trade-off of primary cytology screening with triage compared with primary HPV screening. Nonetheless, the high sensitivity of cytology for high-grade lesions was associated with the prevalence of precancerous lesions in our study and would be significantly lowered in the population-based screening (24).

Increasingly, studies are confirming and supporting AI technology in cytology-based cervical cancer screening (25, 26). Our previous studies also showed that AI-based cytology was comparable and feasible to manual cytology in the general and referral populations (16, 27). This study further showed that the effectiveness of HPV strategies incorporating AI technology is comparable to that of incorporating manual cytology. Nonetheless, unlike in primary cytology screening (25, 26), the role of AI in the reduction in manual work is limited in HPV-based strategies because many women who are HPV positive have abnormal cytology and need TBS classification by manual work. Further studies are needed to advance AI technology in the automated TBS classification of cytology.

### Limitation of This Study

First, the study could not evaluate strategies in two screening rounds or more because of the cross-sectional design. All women were assessed by colposcopy immediately, which permitted the estimation of immediate sensitivity and specificity. Considering that, the current strategies, including primary HPV screening with triage and co-testing, would acquire lower sensitivity and higher specificity when the women attend the repeating test rather than immediate colposcopy. Second, the referral population in the study is intended to make a robust estimation of the sensitivity because all participants could be evaluated by colposcopy. However, this means that the scenario, e.g., the high prevalence of HPV infection, cytological abnormality, and CIN diseases, may affect the parameter estimations in the population-based screening, particularly specificity. Hence, we used PLR and NLR as accuracy surrogates for clustering patterns instead of sensitivity and specificity because they are integrated indicators including the information of both sensitivity and specificity and are less affected by prevalence.

Unsupervised clustering techniques are sensitive to distance measures, but it is difficult to distinguish whether the identified clusters are underlying structures or are artifacts of sampling variation (28). As such, the results should be treated with caution. The combination of mathematical methods and clinical practice for identifying significant clusters mitigates the risk of instability affecting the lower branches of the dendrogram.

## Conclusions

The study formed five significant clusters in terms of accuracy, cost, and efficiency based on 14 common screening strategies in routine practice. Primary HPV screening with triage of cytology, 16/18 genotyping, or both was nested within a cluster and provided an optimal balance between sensitivity and the number of colposcopies, primary tests performed, the load of follow-up, and manual work compared with the remaining strategies. Our study provided clinical and methodological evidence on the choice of high-performance HPV-based screening strategies.

## Data Availability Statement

The original contributions presented in the study are included in the article/supplementary material. Further inquiries can be directed to the corresponding author.

## Ethics Statement

The studies involving human participants were reviewed and approved by National Center for Chronic and Non-communicable Disease Control and Prevention, China CDC. The patients/participants provided their written informed consent to participate in this study.

## Author Contributions

LW conceived and designed the study and took responsibility for the integrity of the data and the accuracy of the data analysis as guarantors. HBa performed the study and conducted the investigation, analysis, and draft writing. HBi, XZ, YZ, LF, and HW contributed to the investigation, critical revision, and validation. All authors contributed to the interpretation of the results. All authors contributed to the article and approved the submitted version.

## Funding

This study was supported by grants from the Association of Maternal and Child Health Studies (2017AMCHS006) and the National Natural Science Foundation of China (81903328). None of the funding organizations were involved in the design and conduct of the study; collection, management, analysis, and interpretation of the data; preparation, review, or approval of the manuscript; or the decision to submit the manuscript for publication.

## Conflict of Interest

The authors declare that the research was conducted in the absence of any commercial or financial relationships that could be construed as a potential conflict of interest.

## Publisher’s Note

All claims expressed in this article are solely those of the authors and do not necessarily represent those of their affiliated organizations, or those of the publisher, the editors and the reviewers. Any product that may be evaluated in this article, or claim that may be made by its manufacturer, is not guaranteed or endorsed by the publisher.
